# Knowledge transmission, culture and the consequences of social disruption in wild elephants

**DOI:** 10.1098/rstb.2024.0132

**Published:** 2025-05-01

**Authors:** Lucy Bates, Victoria Louise Fishlock, Joshua Plotnik, Shermin de Silva, Graeme Shannon

**Affiliations:** ^1^ School of Psychology, Sport and Health Sciences, University of Portsmouth, Portsmouth PO1 2UP, UK; ^2^ Amboseli Trust for Elephants, Nairobi, Kenya; ^3^ Centre for Ecology and Conservation, University of Exeter College of Life and Environmental Sciences, Exeter TR10 9FE, UK; ^4^ Department of Psychology, Hunter College, City University of New York, New York, NY 10065, USA; ^5^ Department of Psychology, The Graduate Centre, City University of New York, New York, NY 10016, USA; ^6^ Department of Ecology and Evolution, University of California San Diego, La Jolla, CA 92093, USA; ^7^ Trunks & Leaves Inc, Pittsfield, MA 01201, USA; ^8^ School of Environmental and Natural Sciences, Bangor University, Bangor LL57 2DG, UK; ^9^ Norwegian Institute for Nature Research, 7034 Trondheim, Norway

**Keywords:** elephant culture, elephant conservation, social disruption, social knowledge

## Abstract

Cultural knowledge is widely presumed to be important for elephants. In all three elephant species, individuals tend to congregate around older conspecifics, creating opportunities for social transmission. However, direct evidence of social learning and cultural traditions in elephants is scarce. Here, we briefly outline that evidence then provide a systematic review of how elephant societies respond to the loss of potentially knowledgeable individuals or opportunities for knowledge transfer, which we characterize as *social disruption*. We consider observations from 95 peer-reviewed, primary research papers that describe disruption to elephant societies or networks via the removal or death of individuals. Natural deaths were mentioned in 14 papers, while 70 detailed human-caused deaths or disruption. Grouping descriptions according to consequences for behaviour and sociality, and demography and fitness, we show that severely disrupted populations are less cohesive, may exhibit reduced fitness or calf survival and respond inappropriately to threats and predators. We suggest that severe social disruption can inhibit or break potential pathways of information transmission, providing indirect evidence for the role of social transmission in elephants. This has implications for elephant conservation amid increasing anthropogenic change across their habitats.

This article is part of the theme issue ‘Animal culture: conservation in a changing world’.

## 1. Introduction

Evidence for animal culture—defined by Laland & Hoppit as ‘group-typical behaviour patterns shared by members of a community that rely on socially learned and transmitted information’ ([1], p. 151)—is increasingly widespread in the animal kingdom, recognized in taxa as diverse as bees and whales [2–4]. Animals may use socially transmitted knowledge to obtain food, attract mates, learn about predators or threats and acquire appropriate or relevant social behaviour [5]. These socially acquired ‘group-typical behaviour patterns’ can be thought of as ‘traditions’, such that a population’s entire culture can be considered as an array of traditions covering different domains of behaviour [6]. Social learning is, therefore, a requisite of culture, but evidence of social learning alone is not sufficient to claim culture: there must also be evidence that the acquired behaviour persists (for a tradition) and has spread among members of a group (for culture) [7].

Social learning and animal culture can have important implications for conservation [8,9]. For example, accounting for socially transmitted knowledge and behavioural traditions can improve post-release survival of re-introduced individuals [10–12], help to define relevant ecological niches or adaptations [13–15], and determine whether or how animals can acquire important information such as migration routes [16–18]. This relevance to conservation means policymakers, practitioners and researchers have a responsibility to consider social networks and the social acquisition of knowledge in the species they seek to conserve [19]. Yet, it can be difficult to identify, observe and study social learning in the wild [7], which may lead to some species being excluded from advances in conservation practice or policy. Therefore, alternative evidence of the role of social learning and culture may be necessary. Disrupting social systems by removing individuals can sever information transmission chains [20,21]; assessing how animals respond to such disruption may provide indirect but useful evidence of the role of transmission chains and social learning.

Somewhat surprisingly, elephants are one such taxon where it has so far proven difficult to determine the extent of social learning and the role of culture in their societies. There are three recognized elephant species: African savannah (*Loxodonta africana*) and forest (*Loxodonta cyclotis*), and the Asian elephant (*Elephas maximus*). Globally, all three are declining [22–24], and they are listed as endangered or critically endangered [24–28]. Elephants are considered conservation icons, being charismatic keystone species that shape the ecosystems they inhabit [29,30], and there is often an assumption that elephants *should* exhibit culture: they are large-brained, highly social and have long periods of juvenile dependency, which provides plenty of opportunity to learn from elders and peers [6]. However, evidence for the social acquisition and transmission of knowledge has not been previously reviewed. We aim to address this gap by summarizing the evidence suggesting culture may be important to elephants and presenting newly synthesized evidence of what happens when elephant social networks are disrupted by the death or long-term removal of any member of a population.

### (a) Current evidence of culture in elephants

#### (i) Opportunities for social learning

Elephants occupy extensive habitat gradients [31–33], with each environment presenting different ecological challenges that likely require specialized behavioural adaptations (e.g. [34,35]). Moreover, all elephant species exhibit fission–fusion social dynamics [32,33,36–39] that provide ample opportunities for vertical, horizontal and oblique transmission of information throughout their lifespans. A mother and her dependent offspring form the basic unit, and calves remain dependent on their mothers for many years [40–42]. Calves consistently maintain close proximity to elders (mothers or allomothers) [38,43], and although it has not yet been validated as such, this proximity may allow visual and olfactory investigation, functionally equivalent to the peering behaviour of immature primates that serves as an index of their social learning [44].

In savannah elephants, several adult females group together to form a ‘family’ or core group, which is typically led by a matriarch (usually the oldest female) [38]. These groups are characterized by persistent and strong social bonds, although adult females within a core group may separate for hours or days before reuniting [45]. Asian elephants are usually found in smaller groups than savannah elephants, with higher rates of fission and fusion among social affiliates [32,33], but this may be an effect of historical human impacts [33]. Forest elephants likely also range in small groups, and the closest female associations may be less tied to relatives than with savannah elephants [46], as sexually mature females can disperse from their natal group [47]. However, forest elephants are known to associate in large numbers at forest clearings, apparently with the objective of maximizing opportunities for social interaction [36].

In all elephant species, males disperse from the family as teenagers [47–50], gradually developing independence. Young independent savannah males preferentially seek out the company of older males [48,49,51], providing an opportunity to learn male dominance structures, which are based on sexual status, physical size and strength, and behavioural traits [52]. As they age, males of all species enter annual ‘musth’ phases—periods of greatly heightened testosterone that can last several months, which signal social and sexual maturity [53]. During musth, males range widely and compete for sexually receptive females [52,54–56]. However, for most of the year, savannah males are sociable and commonly form all-male groups [49,57,58]. These male groups may be particularly important under conditions of high-risk foraging, for both Asian and savannah elephants [50,59].

Alongside their complex social structure, all three species exhibit an extensive communicative repertoire, using vocalizations and chemical, tactile and visual signals [60–62]. Elephant societies are built on individual recognition: savannah elephants recognize and respond appropriately to many other individuals across vocal and olfactory domains [63–65] and communicate intentionally [66], targeting vocalizations towards particular conspecifics using individual identity labels [67]. Furthermore, experimental and observational studies have shown that elephants are empathic and understand the goals of others [68–70], that they exhibit complex responses to dead conspecifics [71–76], and that Asian elephants have the capacity for self-awareness [77]. These cognitive abilities might support or contribute to forms of social learning [78] (but see also [79]).

#### (ii) Evidence for social learning and information sharing

Captive savannah elephants presented with a classic ‘two-action’ feeding apparatus did not copy a demonstrator, but results provided evidence of local enhancement (whereby the actions of the demonstrator likely focused the attention of the learner on a particular location) [80]. Similarly, captive Asian elephants displayed stimulus enhancement (where attention is focused on an object) in response to apparatus set up for the ‘Aesop’s fable’ experimental task, but there was no evidence they learned to solve the task by watching the actions of others [81]. Captive Asian elephants in Thailand cooperated in an experimental task requiring coordinated rope pulling [69] and captive individuals in Myanmar tested on a similar task in larger groups worked to mitigate competition to maintain a high level of cooperation [82]. This cooperation could be based on social affordance learning, where the operating characteristics of objects are shared. There is evidence of vocal imitation in both savannah and Asian elephants, whereby they copy sounds they hear that are outside the normal vocal repertoire of the species [83,84]. Finally, observational data suggest that older female savannah elephants *might* actively demonstrate (i.e. ‘teach’) appropriate oestrous behaviour to naive females as they become sexually receptive for the first time, so that they attract the best males [85].

Brakes *et al*. [9] encourage consideration of indirect evidence for social learning. Risky foraging strategies may be socially influenced; male savannah elephants are more likely to be crop raiders if their closest associates are raiders [59], and the acquisition of fence-breaking behaviour might be similarly socially transmitted, with researchers currently testing this prediction (V. L. Fishlock 2024, personal communication; J. Plotnik 2024, personal communication). An experimental study assessing differences in reactions to cues of a potential danger (the scent of cloth worn by Maasai warriors, who occasionally spear savannah elephants) showed families with no direct history of being speared reacted as strongly as families that had experienced spearing [86]. It remains to be tested if this response is based on social acquisition.

#### (iii) Evidence for socially acquired traditions or culture

Despite the relative paucity of evidence for social learning, there are some compelling indications of persistent traditions in free-ranging elephants. Familial social network positions may be traditional, with network analysis demonstrating that immature savannah females attempt to assume and maintain the place of their mothers and family in society after older individuals were killed by poaching [87]. In forest elephants, travel paths and resource patch selections appear to be traditional, as these elephants use well established paths to specific resource patches, and their preferences are not explained by resource quality or accessibility [88]. Social acquisition seems highly likely in both cases, although it is yet to be definitively demonstrated.

Moreover, evidence suggests savannah elephants may possess something akin to vocal dialects, which are potentially socially learned and traditional. Comparisons of combinatorial vocalizations produced by the three different elephant species found sequence differences among populations’ repertoires that did not align with phylogenetic distance [89], and a recent analysis of ‘rumble’ vocalizations across savannah elephants in Amboseli and Samburu found differences in the fine structure of the calls both between and within the populations [90]. Group membership was a better predictor of rumble similarity than relatedness, suggesting the call features were learned socially rather than genetically inherited.

#### (iv) Assessing the consequences of social disruption

Social disruption is known to have impacts for behaviour and conservation [20], although it is difficult to study these effects in wild populations [91,92]. Evidence suggests that social network structures can be resilient to severe social disruption across many animal species [87,91,93], but individual responses can vary and remain poorly understood [94]. Throughout their ranges, elephant populations have been subject to considerable social disruption, both from natural events (e.g. severe droughts) [95,96] and from anthropogenic causes (e.g. poaching or hunting; accidents such as train collisions; and management interventions such as culling, translocation or euthanasia after conflict arises) [97–99]. Reviewing available literature on elephant ecology and management provides an opportunity to broaden our knowledge of the consequences of social disruption and to consider its impacts on knowledge transmission.

## 2. Methods: review of social disruption and its consequences in elephants

This systematic review was conducted following established procedures used in medical sciences [100,101].

### (a) Inclusion criteria

To be included, papers had to:

(1) Be published in peer-reviewed, English-language journals. Book chapters and grey literature were excluded;
(2) Relate to free-ranging elephant populations. Studies of captive or semi-captive elephants were excluded;
(3) Include an element of direct behavioural data collection (observational or experimental). Studies with a veterinary, genetic or morphological focus, and studies that *only* used remote sensing data collection techniques (such as dung counts, aerial counts and GPS/radio collar tracking) without any direct observations were excluded on the grounds that they could not attest to the behavioural implications of any social disruption;
(4) Detail some form of social disruption. This could be the death or removal (i.e. translocation) of one or more elephants of any age within the group or population being studied. Papers were retained if they contained any of the words (or variations of): death, culling, hunting, lethal population control, poaching, orphaning, removal or translocation.

Criteria 1 and 2 were imposed in the initial stages of the search, criterion 3 was enforced when paper titles and abstracts were screened, and criterion 4 during full-text screening and data extraction.

Reports of birth control were not included *a priori*. Birth control aims to alter the demographic profile of a population, which could be considered disruptive, but it does not do so by removing existing elephants. Additionally, short-term or immediate disturbance of an elephant group owing to a finite activity (e.g. tourist viewing or crop-protection activities) was not included in our definition of disruption unless it resulted in long-term change to the demographic composition of the elephant group.

### (b) Database searches

We searched Web of Science and Science Direct using the search terms Elephant AND Loxodonta OR Elephas, with a date range of 1970–2023. For Google Scholar, we used the Advanced Search function to exclude studies with the words ‘captive’ or ‘captivity’. For the Web of Science and Science Direct searches, excluding captive studies in the search proved unreliable, so we manually excluded studies of captive elephants from those searches.

**Figure 1.**
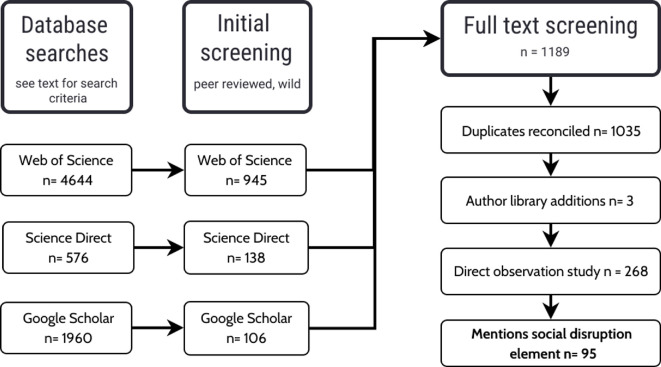
The systematic review process.

These searches, conducted in January 2024 and detailed in figure 1, returned 4644 papers from Web of Science, 576 papers from Science Direct, and 1960 from Google Scholar, the total being reduced to 1189 papers after applying inclusion criteria 1 and 2. These were uploaded to a Rayyan.ai database. Rayyan is a Web-based application, which was used to manage the subsequent manual screening and identify duplicates, which were checked and resolved by author L.B. This resulted in a database of 1035 citations detailing studies of wild elephant behaviour.

### (c) Screening

L.B. screened the titles and abstracts in Rayyan for inclusion criterion 3, then manually screened the full text of the remaining 268 papers using search terms in criterion 4 to retain only those papers that detailed a form of social disruption to a wild population. L.B. then scanned her own research library to ensure inclusion of any other papers that met all the inclusion criteria. This step added three papers, as detailed in figure 1 and the electronic supplementary material.

### (d) Data extraction and assessment of bias

Metadata from the 95 retained papers were entered into a spreadsheet, noting the elephant species and population studied, study country and study type (e.g. observational, experimental). We recorded the type of disruption documented in the paper (e.g. natural death; human-caused death, incorporating poaching, culling, hunting, conflict killing and accidents; unspecified deaths; or translocations) and details of the number and age/sex of the individuals affected, summarized any socio-behavioural and demographic or fitness consequences presented, and noted evidence of behavioural or social resilience, age or experience effects, or suggestions of increased conflict with humans.

Assessing possible sources of bias is an important part of reviews [100,101]. It is particularly important to be aware of publication bias, where positive or strong results are more likely to be reported. We consider this in §3.

### (e) Analysis

Extracted data were grouped according to the type of disruption experienced by the elephants. We then considered the pattern of consequences reported, grouped as *behavioural and social impacts* and *demographic or fitness consequences*. Finally, we considered the impacts for behavioural or group resilience, any age or experience effects that were recorded, and whether any likely impacts for people were noted (e.g. conflict or coexistence).

## 3. Results and discussion

This review surveyed 95 peer-reviewed research papers detailing long-term disruption to the social structure of elephant populations. Papers represent elephants from 12 countries, as shown in table 1, with research observations heavily concentrated in a few places. Most papers (*n* = 81, 85%) reported effects for savannah elephants, with just *n* = 8 describing Asian elephants and *n* = 6 for forest elephants. The full dataset of observations mined from each paper can be found in the electronic supplementary material, while table 2 summarizes the extracted observations.

**Table 1.** Source of extracted data, showing the number of papers per species, country and type of disruption. Some papers mention more than one type of disruption. ‘Orphans’ refers to papers that discuss the fate of orphans without stating how the mother died. ‘Culling and translocation’ refers to elephants that were spared from culling operations and subsequently translocated to establish new populations as juveniles. Extant resident population information is taken from *IUCN Red List* assessments [24–28].

species	countries with extant resident populations	countries contributing papers	country	number of papers	disruption types described
Asian (*n* = 8)	13	2	India	4	hunting, poaching, train accidents, unspecified
			Sri Lanka	3	conflict killing, translocation, unspecified
			combination	1	unspecified
forest (*n* = 6)	20	3	Central African Republic	3	poaching (*n* = 3), hunting
			Gabon	1	natural death
			Republic of Congo	1	poaching
			combination	1	poaching
savannah (*n* = 81)	23	7	Kenya	37	conflict killing (*n* = 3), natural death (*n* = 7), orphans (*n* = 5), poaching (*n* = 13), translocation (*n* = 5), unspecified (*n* = 7)
			Tanzania	11	natural death, poaching (*n* = 10)
			Uganda	2	natural death, poaching (*n* = 2)
			Botswana	3	natural death (*n* = 2), translocation
			Namibia	1	poaching
			South Africa	25	culling and translocation (*n* = 17), hunting, natural death, poaching, translocation (*n* = 4), unspecified
			Zimbabwe	1	culling
			multiple	1	unspecified

**Table 2.** Reported effects of social disruption. Human-caused deaths incorporate culling, poaching, hunting, conflict killing and accidents. Some papers mention more than one type of disruption.

		natural deaths			human-caused deaths			translocation		unspecified	
		Asian (*n* = 0)	African forest (*n* = 1)	African savannah (*n* = 13)	Asian (*n* = 4)	African forest (*n* = 5)	African savannah (*n* = 48)	Asian (*n* = 1)	African savannah (*n* = 12)	Asian (*n* = 3)	African savannah (*n* = 11)
evidence comments on	sociality/behaviour	—	1	13	3	4	46	1	12	3	9
	demography/fitness	—	—	7	2	4	37	1	4	—	5
data suggest	resilience in behaviour, sociality or fitness	—	—	1	1	—	20	—	6	—	2
	age/experience effects	—	—	4	—	—	18	—	1	—	5
	potential for increased human conflict	—	—	—	1	1	8	1	4	—	—

The initial search terms allowed inclusion of papers from 1970 to 2023, yet only three papers from the final 95 were published prior to 2000 (all three of which detail observations of savannah elephants in Kenya). Since 2000, the rate of papers reporting social disruption appears consistent: 34 relevant papers (36%) are from 2000 to 2009, 39 (41%) are from 2010 to 2019, and 19 (20%) are from the 4 years up to the end of 2023. Papers variously reported disruption effects on a handful of individuals (minimum = 1 elephant) up to entire populations, covering all age- and sex-classes of all three elephant species. The disruption type most frequently mentioned was poaching (*n* = 33), followed by ‘culling and translocation’ (*n* = 17, referring to elephants that were spared from culling operations in southern Africa as juveniles and translocated to establish new populations) and natural deaths (*n* = 14). Cause of death was not specified in 12 papers.

### (a) Consequences of social disruption

It is important to acknowledge that individual- and population-level consequences of natural and human-caused disruption have not yet been widely reported for Asian and forest elephant species, as is evident from tables 1 and 2. However, social disruption appears to have substantial effects on elephants. In all three species, demographic and fitness consequences were most often reported after the killing of elephants by humans (particularly poaching). This can skew natural sex ratios, which reduces mate competition and thereby potentially reduces the fitness of the remaining elephants [47,102–111]. Social–behavioural consequences were also widely reported, with evidence from savannah elephants that social disruption can reduce social cohesion, changing the social structure of populations and rendering individuals less socially discriminating [45,104,105,112]. Reductions in social interaction after disruption were also noted in Asian elephants [33].

Reduced social cohesion was noted in savannah elephants after the natural death of a matriarch [39,113,114], but the most profound changes reported occurred after more extensive, human-induced disruption (i.e. poaching and culling) or severe droughts [105,107,109,115–118]. This is likely to be due to the scale and extent of deaths caused by such disruption events, whereby a considerable proportion of a population perishes in a relatively short time span. Translocation can also have severe negative impacts for the individuals removed [119–121], although the social and fitness consequences have not been widely studied for the source and receiving populations [122]. However, where the scale of translocation is small, the consequences may be less pervasive than for extensive disruption such as poaching.

Even among severely disturbed populations, the consequences of social disruption can vary. In cases of population-wide intense or long-term poaching, such as at Dzanga, Ruaha and Mikumi [47,105,109], age of primiparity and inter-birth intervals are high, resulting in slow population recovery (although fecundity in forest elephants may reflect generally slower life history [47]). In other poached savannah populations, reproductive rates increased following disruption—as seen in Samburu and Tarangire [123,124]. In the much smaller, spatially and socially limited populations of South Africa that were founded by translocating juveniles spared from culling exercises [125], reproductive output is very high, with very low ages of primiparity and short inter-birth intervals resulting in exponential population growth [126–128].

These patterns may not be as contradictory as they appear. Disruption in Mikumi, for example, was extremely severe, with very few older adults remaining after devastating poaching and extremely low sociality among survivors [104]. We suggest the young age and weak social bonds could have impeded reproduction. Poaching in Samburu and Tarangire—although severe—was not as catastrophic, which perhaps allowed a slightly greater degree of cohesion and resilience amongst survivors [124,129,130], which in turn could enable more successful reproduction. Similar social buffering of disruption effects has been noted in mountain gorillas, where relationships in cohesive social groups mitigated the long-term adversity typically expected after loss of the mother [131]. For elephants, decreased feeding competition in recovering populations could also contribute to mitigation and increased reproduction rates, although this was evidently not the case in Ruaha and Mikumi. High reproductive rates have been recorded in Kruger after culling [132], where remaining core groups were left largely intact and resource competition was reduced. Alongside access to nutritional resources, sufficient social cohesion to allow for something approximating a normal social structure appears important for the resumption of successful reproduction.

Although high reproductive rates were reported in Samburu, survivorship remained low, particularly compared with life expectancy in Amboseli, which has a population with many more elders, having been spared the intensity of poaching observed elsewhere in Kenya [123]. This points to the potential role of elders in increasing population life expectancy, consistent with observations from Amboseli and semi-captive Asian elephants (not included in the review) that calf survival increases when older females are present in the family [38,41,133]. Moreover, maternal and matriarchal loss are the strongest predictors of life expectancy in Amboseli [41,113]; calves of primiparous savannah females experience the highest risk of mortality [41,42,113]; and the presence of a mother reproducing simultaneously with her daughter improves survival of the daughter’s calf [41]. Neither age nor size of the mother at first birth influence chances of calf survival, which suggests this increased mortality is not about the mother’s physicality (nor her ability to produce sufficient milk). Instead, it is possible that mothers have to learn key maternal skills, such as how to manage and balance their own needs with those of their calf [41], and that modelling from more experienced individuals enhances this skill acquisition. However, the exact contribution of knowledge to survivorship—and the role of matriarchs and elders in sharing this knowledge—remains to be tested.

### (b) Implications for knowledge acquisition and culture

While further research is required to determine how elephants acquire and share information, several studies clearly demonstrate that age and experience are important to savannah elephants, as they are in other long-lived species [134]. Acquired knowledge can be remembered over significant timeframes by elders [64], and savannah families led by older matriarchs display greater social knowledge [117,135], more appropriate behaviour in response to threats and predators [136,137] and greater calf survival during droughts [96]. Moreover, even in normal climatic periods the presence of matriarchs is important for calf survival [113], and the absence of elders might contribute to slow calf recruitment in some populations after severe disruption [107]. These matriarch effects may not be based specifically on knowledge, however, but in some cases could reflect the age-and-size-based dominance hierarchies of savannah elephants that potentially allow families led by older matriarchs to secure access to better resources [138]. More research is required to differentiate these possibilities.

While elders may be more knowledgeable, learning and adaptations to new problems or extreme situations can be slow, exemplified by observations of increasing predation by lions in the Savuti region of Chobe, Botswana [139,140]. Other ungulates migrate away from the area in the dry season, reducing prey abundance for resident lions. However, artificial water points have been added relatively recently, and these are monopolized by elephants in the dry season. This has allowed elephants to be more sedentary, and lions have taken advantage of this by successfully hunting (mostly juvenile) elephants [139,140]. When the initial studies were published, there was no evidence that elephants were adapting to the increased predation risk by altering their behaviour (e.g. becoming more vigilant or clustering together). However, large lion prides targeting elephants are rare, so learning about this specific threat could be slow, making elephant decision-making appear maladaptive in the short term. Indeed, the overall threat from lions is likely to remain relatively low at the population level and, coupled with the elephants' conservative behavioural patterns, this could cause delayed responses to such environmental pressures [141]. Follow-up studies would provide valuable insights into whether these elephants have since altered their behaviour.

Moreover, while elders are important in elephant society, groups led by younger individuals are not necessarily bound to make poor choices. When forced, elephants can adapt and learn to survive with reduced social structures or young populations, as seen in the responses of some heavily poached populations. With fundamental decisions relating to foraging and mating, there is considerable evidence that youngsters can function nearly as well as elders [106]. When elders are not present, elephants rely on associating with age-mates [142,143] and one potential benefit of this, beyond the typical socio-ecological benefits of grouping, could be the horizontal sharing of knowledge. Juveniles work to maintain the social ties and network structure of their deceased core-group members [87], although this does not always appear to be possible [104]. Perhaps social cohesion patterns *before* poaching are important, as well as the number of age-mates remaining with which to form a network after disruption [143,144].

Even after extreme disruption—as in the cases of orphans from culling events being used to establish new elephant populations in South Africa, where the juvenile elephants had no access to elders as they matured—it is apparent that basic biological functions are maintained [126,145]. Indeed, many of these populations have exhibited exponential growth rates, despite the lack of elders [127,128]. However, population numbers do not tell the whole story. Some of these ‘cull-orphan’ populations lack potentially significant forms of knowledge that are acquired and used by elders in other, intact, populations—such as fine-scale social discrimination and interpretation of predatory threat [117,137]; and there are several reports that some of these populations exhibit aberrant or deviant behaviours such as heightened aggression that can endanger people, other animals and themselves [97,127,146]. It is reasonable to predict that this behaviour stems from the lack of appropriate role models and may additionally reflect responses to long-term trauma or early life adversity from losing their mothers during the cull [147–149].

Furthermore, it should be noted that artificial water points that allow year-round access to water are typical of the small, fenced game reserves of South Africa that have exhibited exponential growth rates [97]. The reproductive success of the translocated cull orphans in these reserves is perhaps possible—or at least rendered more likely—because it may be easier for the young ‘matriarchs’ in these juvenile populations to individually acquire necessary resource information, with long-term knowledge of resource locations likely being less critical in such relatively limited areas with abundant and predictable food and water. Conversely, translocations that have moved elephants to more expansive new areas often result in homing behaviour [119–121,150,151], and learning about new areas can be slow and cautious when the range area is potentially extensive [122,152].

### (c) Limitations and consideration of bias

A number of limitations and biases are inherent to a review of this nature. Firstly, research is not evenly spread across species and populations. Most of the results describe savannah elephants in Kenya (where there are two long-term research sites [153,154]) and South Africa (where much research has focused on the ecological impacts of fencing elephants in small reserves [125]). The relative under-representation of forest and Asian elephants must be considered when drawing conclusions—especially given subtle differences in social systems across the species. However, the relative lack of evidence about forest or Asian elephants does not imply that social disruption and the loss of elders are not important for these species: some studies suggest that forest and Asian elephants respond like the savannah species to social disruption [33,47,108]. Although more research is needed, for now we should assume that social disruption will have similar fitness consequences in all elephants.

Secondly, we only included peer-reviewed papers that contain direct observations of elephant behaviour and which note some form of social disruption to the population or study group. It is likely that more evidence could be gained from widening the search parameters to include grey literature or studies that only involved remote data collection rather than direct behavioural observations. However, while this may add to the depth of evidence, we do not think it would alter the general direction of the results presented here. Thirdly, it is not easy to determine the causes of many of the effects noted here, particularly where papers mention two or more types of disruption, or in the case of ‘culling and translocation’ (table 1 and electronic supplementary material)—where either or both actions can be considered highly disruptive and potentially traumatic [97,98,147].

Most papers studied the social structure and/or demographics of the population after a disruption, but not necessarily individual behavioural responses. This reflects the substantial research effort undertaken to understand elephant demography and general socio-ecology, perhaps at the cost of understanding individual differences [155], and may reflect a publication bias against ‘case studies’ of individuals. Additionally, relatively few studies included here directly discussed the consequences of disruption for learning and knowledge acquisition, with little mention of observed resilience or age-experience effects, nor of how the disruption may impact humans living or working alongside the elephants. This needs addressing in future research, given increasing pressure between humans and elephants [156–158], and the acknowledged role of social learning in conservation success [8,9]. We have sought to be transparent when our consideration is based on a degree of interpretation and we recognize that our predictions need testing and verification.

### (d) Conclusions and implications

Although evidence of culture remains relatively sparse for elephants, data demonstrating socially inherited behavioural traditions are building, as we have outlined. Moreover, the maxim that elephant elders ‘are repositories of knowledge’ [135] holds up: this conclusion, previously drawn from a few seminal studies (e.g. [96,135,136]), is widely apparent across the papers reviewed here (and see [159]). We cannot yet conclude *how* elders acquire their knowledge, nor whether naive individuals learn by observing them, but it is clear that elephants benefit from having elders present in their society. However, further research is needed to explore the extent of social learning and the persistence and spread of traditions in elephants.

Given the relative paucity of studies researching elephant culture, we adopted an alternative approach—assessing the effects of disrupting social structures. Social disruption, particularly when it is extensive, evidently has substantial consequences for elephants. Disruption reduces social cohesion, and although there is some resilience and elasticity to elephant sociality, disturbance can result in severe, long-term fitness consequences across populations and generations, with some evidence that this may have downstream impacts for people in close proximity to elephants. While elephant societies can function at a basic level without elders, maladaptive or deviant behaviour can arise. Social structure can influence information spread and, in at least some of the research evidence reviewed here (e.g. [115,117,121,137,152,160,161]), it is likely that social disruption had substantial impacts because it severed potential information transmission chains, thus providing indirect evidence of the role of social learning in elephant society. Our approach may be beneficial for other species where studies of social learning are lacking, to ascertain whether conservation efforts should address disruption to social networks.

Based on our review, we argue that future elephant conservation plans should prioritize social cohesion and preservation of group structure: maintaining existing vertical, oblique and horizontal transmission chains should be a primary aim of management efforts.

Additionally, we recommend (i) prioritizing disruption assessments for forest and Asian elephants, which are currently under-represented; (ii) clearly reporting the type of disruption experienced by populations being studied and increasing consideration of any resilience or age–experience effects, as well as noting if any increased conflict with people is likely; (iii) ensuring that translocation strategies assess both source and destination populations and incorporate follow-up monitoring; and (iv) ensuring management and permitting authorities consider the behaviour, demography and cultural history of elephant populations when determining appropriate actions.

All three elephant species share much of their range with humans, and our transformations of their habitats and society could have profound consequences for their survival [162] as well as our wellbeing [163]. We must consider how elephants may respond or adapt [158,164,165], and that requires taking potential knowledge acquisition pathways and social consequences into account.

## Data Availability

The electronic supplementary material, available online, includes details of all papers that were included in this systematic review, and shows the information we extracted from each [166].
